# Long-distance transmission patterns modelled from SNP barcodes of *Plasmodium falciparum* infections in The Gambia

**DOI:** 10.1038/s41598-019-49991-4

**Published:** 2019-09-18

**Authors:** Alfred Amambua-Ngwa, David Jeffries, Julia Mwesigwa, Aminata Seedy-Jawara, Joseph Okebe, Jane Achan, Chris Drakeley, Sarah Volkman, Umberto D’Alessandro

**Affiliations:** 1 Medical Research Council Unit The Gambia at London School of Hygiene and Tropical Medicine, Banjul, The Gambia; 2 Harvard School of Public Health, Boston, Massachusetts, USA; 3 London School of Hygiene and tropical Medicine, London, UK

**Keywords:** Population dynamics, Parasitic infection

## Abstract

Malaria has declined significantly in The Gambia and determining transmission dynamics of *Plasmodium falciparum* can help targeting control interventions towards elimination. This can be inferred from genetic similarity between parasite isolates from different sites and timepoints. Here, we imposed a *P*. *falciparum* life cycle time on a genetic distance likelihood model to determine transmission paths from a 54 SNP barcode of 355 isolates. Samples were collected monthly during the 2013 malaria season from six pairs of villages spanning 300 km from western to eastern Gambia. There was spatial and temporal hierarchy in pairwise genetic relatedness, with the most similar barcodes from isolates within the same households and village. Constrained by travel data, the model detected 60 directional transmission events, with 27% paths linking persons from different regions. We identified 13 infected individuals (4.2% of those genotyped) responsible for 2 to 8 subsequent infections within their communities. These super-infectors were mostly from high transmission villages. When considering paths between isolates from the most distant regions (west vs east) and travel history, there were 3 transmission paths from eastern to western Gambia, all at the peak (October) of the malaria transmission season. No paths with known travel originated from the extreme west to east. Although more than half of all paths were within-village, parasite flow from east to west may contribute to maintain transmission in western Gambia, where malaria transmission is already low. Therefore, interrupting malaria transmission in western Gambia would require targeting eastern Gambia, where malaria prevalence is substantially higher, with intensified malaria interventions.

## Introduction

The malaria burden in The Gambia has significantly declined and malaria transmission which is markedly seasonal (July-December) has become increasingly heterogeneous, with two main transmission strata—low in the west and moderate to high transmission in the east^1^. *P*. *falciparum*-infected individuals, probably infected during the previous transmission season, can restart the seasonal peak by infecting the local vector^2^. Such a transmission dynamic is probably not entirely local as human movements may contribute to it^3^. Human migration promotes parasite mixing, potentially impacting on the effectiveness of control interventions^4^. The resulting patterns of parasite flow and transmission^5^, genetic diversity and recombination (outcrossing) of different parasite types can be detected using new high-throughput molecular technologies and detailed field epidemiology^6^.

Single nucleotide polymorphism (SNP) barcodes have been developed for *P*. *falciparum* and *P*. *vivax*^7–9^. Similar barcodes have been explored for establishing sibling or ancestral relationships between isolates, from which transmission paths can be inferred^10,11^. Specifically, a 24 SNP barcode for *P*. *falciparum* has enabled genetic fingerprinting, the determination of infection complexity and changes in transmission pattern^12,13^. It can be used to track the spread of clones during transmission and to determine whether an infection is local or imported^5,14^. However current phylodynamic methods applied to prokaryotic pathogens may be inaccurate for reconstructing transmission trees for a complex recombining eukaryote like *P*. *falciparum*. Indeed, recombination and other evolutionary events can exaggerate genetic distance, calling the need for new models to infer transmission links and direction from small SNP barcodes. Integrating high resolution epidemiologic surveys with genetic evidence can allow for more accurate reconstruction of transmission pathways. This will provide important information to understand ongoing residual transmission despite high coverage of control interventions.

## Results

### Populations and genetic diversity

**Figure 1:**
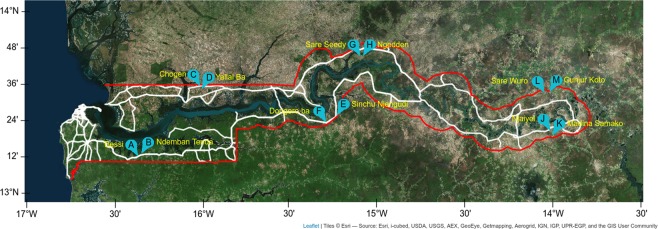
Satellite vegetation map of The Gambia (red boundary) showing study sites. Roads connecting sites are shown in white. Pairs of sites representing two village clusters are called-out alphabetically, with names of the main village shown. Map was created using Leaflet for R. Satellite image is the standard ARCGIS online imagery, sources: Esri, DigitalGlobe, GeoEye, i-cubed, USDA FSA, USGS, AEX, Getmapping, Aerogrid, IGN, IGP, swisstopo, and the GIS User Community. Map was produced on 29th May 2019; https://www.arcgis.com/home/webmap/viewer.html?url=https%3A%2F%2Fserver.arcgisonline.com%2Farcgis%2Frest%2Fservices%2FWorld_Imagery%2FMapServer&source=sd. ArcGIS version 10.3.1 (ESRI, Redlands, CA, USA) was used to map and visualize spatial positions of sites. Topographic data were obtained from DIVA-GIS Free Spatial Data (http://www.diva-gis.org/gdata) and ESRI (http://www.arcgis.com/home/item.html?id=30e5fe3149c34df1ba922e6f5bbf808f). Roads were obtained from Roads^©^ OpenStreetMap contributors and available from https://www.openstreetmap.org through https://overpass-turbo.eu/

In the 2013 malaria transmission season, 1113 *Plasmodium falciparum* isolates were detected by varATS PCR from 12 villages across The Gambia (Fig. 1, Supplementary Fig. 1). Genotypes were successfully obtained for 418 (37.5%) isolates at 54 SNP loci by sequenom. Of these, 355 genotypes were retained to constrain isolate missingness to not more than 12 loci (22%). These samples had a median SNP call rate of 94.5%. The minor allele frequencies of the SNPs ranged between 0.1 to 0.49 for all regions combined. The overall level of heterozygosity for all loci and all populations was 0.424 while overall differentiation between isolates grouped by villages was 0.03 (Weir and Cockerham’s *F*_ST_). When including all isolates and markers, genome-wide index of association between allelic states (I_AS_) was 2-fold higher in western than eastern Gambia.

Mixed allele calls represented 6.2% of retained SNPs called. The proportion of mixed allele calls for SNP loci ranged from 0% to 44%. SNP loci with mixed allele calls increased from west to east, with the highest median of 7% of loci in eastern villages. Similarly, the complexity of infection (COI) modelled from the mixed allele calls with McCOIL ranged between 1–5 clones per infection and increased from west to east (Supplementary Fig. 2), reflecting increasing transmission intensity. The proportion of multiclonal infections increased from 5% at the beginning of the transmission season (June/July) to 32.9% in November, at the peak of transmission.

### Spatiotemporal genetic similarity of *P*. *falciparum* isolates

**Figure 2:**
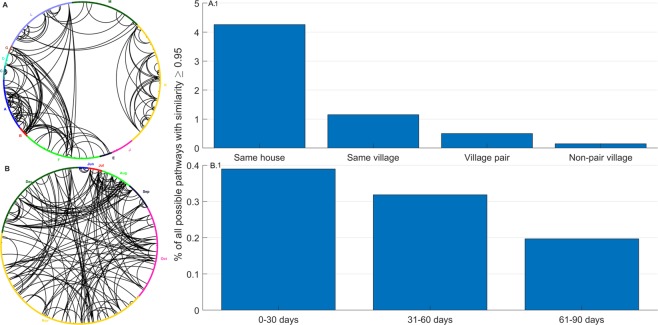
Unidimensional-scaled circular plots of barcode similarity (1 – p-distance) between isolate pairs. Isolates from each site or month are represented by points of a unique colour. Lines link isolates with at least 95% barcode similarity. (**A**) The isolates within each village (identified by the letters outside the circle and distinct colours) have been ordered by a one-dimensional scaling calculated from the donor’s household latitude and longitude. The villages have been arranged around the circle to represent the relative position on The Gambia map. (**B**) Similarity between barcodes collected over time from June to December. Isolates are positioned according to the date of sampling ordered in a clockwise manner for days within a month and months of the year.

We identified 178 pairs of isolates with at least 95% SNP barcode similarity across the transmission season and between villages (Fig. 2, Supplementary Fig. 3; heatmap of all pairwise distances). High proportions of dissimilar barcodes were evident within eastern villages (K, L and M), where malaria prevalence is higher (Fig. 2A). In contrast, isolates from the A and B village pair in western Gambia and from villages G and F in central Gambia shared similar barcodes, suggesting similar clones transmitted between these sites. Genetic similarity (pairwise allelic similarity >95%) was also spatially hierarchical, strongest within households, then within village, and least within non-paired villages (Fig. 2A.1). Infections collected at different months throughout the season were highly similar (Fig. 2B). However, temporal structure was weaker than geospatial structure as only 0.4% of isolates had pairwise barcode similarity beyond 95% within 30 days (Fig. 2B.1). This proportion dropped to 0.2% for samples with a sampling time difference beyond 60 days. Overall, pairwise genetic distance showed a weak increasing trend with days separating samples, only detectable for samples separated by more than 120 days (Supplementary Fig. 4). The relationship between pairwise genetic distance, time and place of sampling was heterogeneous (Supplementary Fig. 5), reflecting the hierarchical experimental design. Hudson’s identity-by-descent (IBD) index detected stronger local pairwise IBD, which linearly correlated with genetic similarity, once the latter was below 55% across the barcode (Supplementary Fig. 6). Given the low density of SNPs per chromosome, genetic distance was used as a measure of relatedness and analysis of the likelihood of transmission pathways for samples was restricted to a genetic distance of 30% or less. Beyond a genetic distance of 30%, pairs of samples can differ by a wide range of evolutionary distances (0.5–3.5 substitutions per sites), (Supplementary Fig. 7), probably due to recombination and other evolutionary events that may exaggerate genetic distances between isolates with a common ancestry.

### Transmission events and probability model

**Figure 3:**
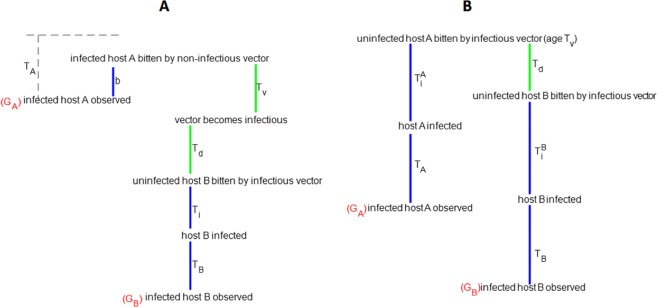
Schematic representation of the stochastic processes involved in directional transmission events constrained by time for malaria parasite life cycle events. (**A**) The six processes indicated for host G_A_ infecting host G_B_ in a transmission event; together leading to a difference between the sampled genotypes accumulated over time. It is assumed individuals were bitten b days before they were sampled and genotyped. The periods b, T_i_ and T_B_ (blue lines) occur in humans, while T_V_ and T_d_ (green lines) represent the time for the vector to become infectious and to infect an uninfected individual, respectively. (**B**) Processes involved in a common source of infection in hosts G_A_ and G_B_ observed at two different times after original transmission event. T_A_ and T_B_ represent the human infectious time since the previous sampling. T_i_^A^ and T_i_^B^ are the times from a mosquito bite to establishment of peripheral parasites.

**Figure 4:**
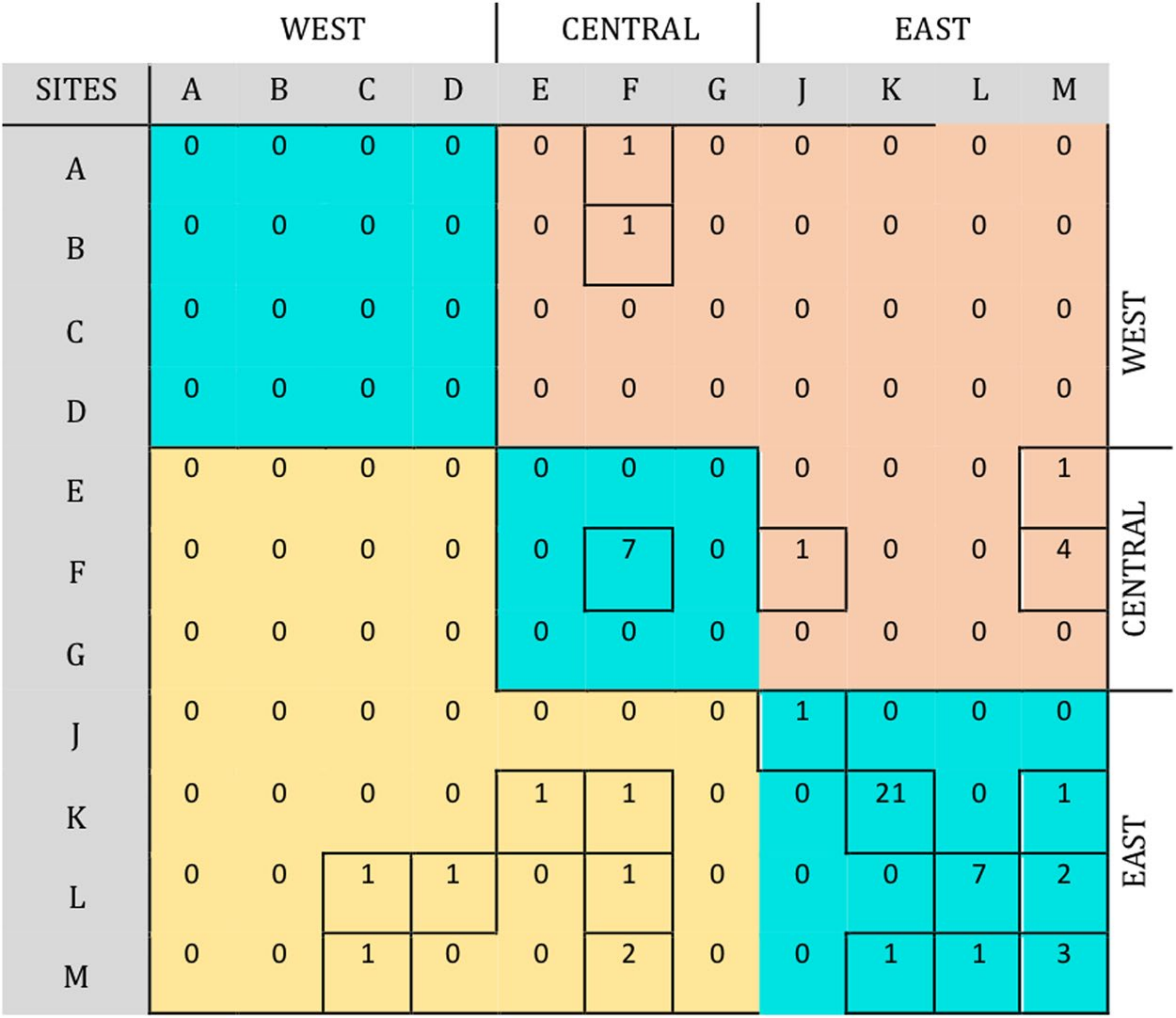
Number of likely person-to-person transmission events by village (60). Cyan indicates transmission within a region [West (A–D), Central (E–G), East (J–M) Gambia]. Brown indicates West to East flow; Yellow depicts East to West flow of transmission paths.

**Figure 5:**
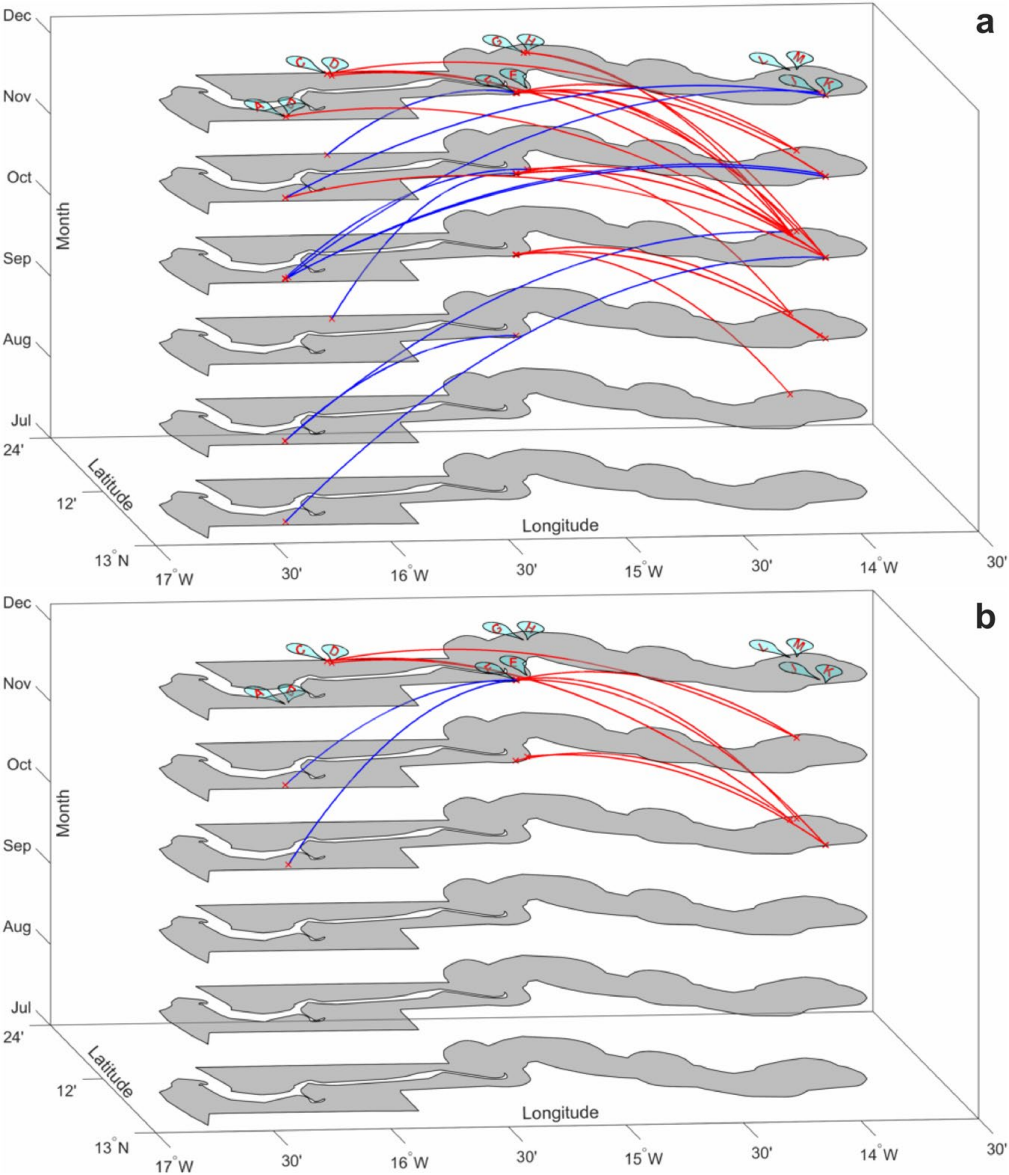
Maximum likelihood transmission paths from most distant westerly and easterly sources. Each arc represents a predicted transmission event (source to sink) colour coded to be red for paths from East to West and blue for West to East. (**a**) All pairs of likely transmission events and (**b**) transmission events constrained by travel for at least one member of the pair in a transmission path.

Based on the time required for *P*. *falciparum* life cycle events shown in Fig. 3, between 32 and 67 days was estimated by simulation for a full person-to-person infection path for chronologically classified infection pairs. The transmission modelling identified 136 person-to-person transmission events, but when this was constrained by contact or travel history the number of pathways reduced to 60. At the village level, these are summarised in Fig. 4, where rows represent the source of a transmission pair and columns the sink of infected individuals. Most transmission paths (65%) occurred between members of the same households or within villages. The higher transmission in eastern Gambia resulted in more than half of all the pathways identified being within the eastern village clusters of J, K, L and M. Village K in the east had the highest number of within village paths. A primary objective of the study was to identify transmission pathways from sources arising in the extreme eastern and western village clusters. Figure 5a,b show the pathways and the direction of infection for these sources, without and with travel history restrictions, respectively. For the travel constrained pathways there were 2 transmission pathways from the most distant western (A-D) villages to central (E-H) villages, while there were 8 pathways from the most distant eastern to central and western villages.

### Multiple infections from a single source- ‘super-infectors’

**Figure 6:**
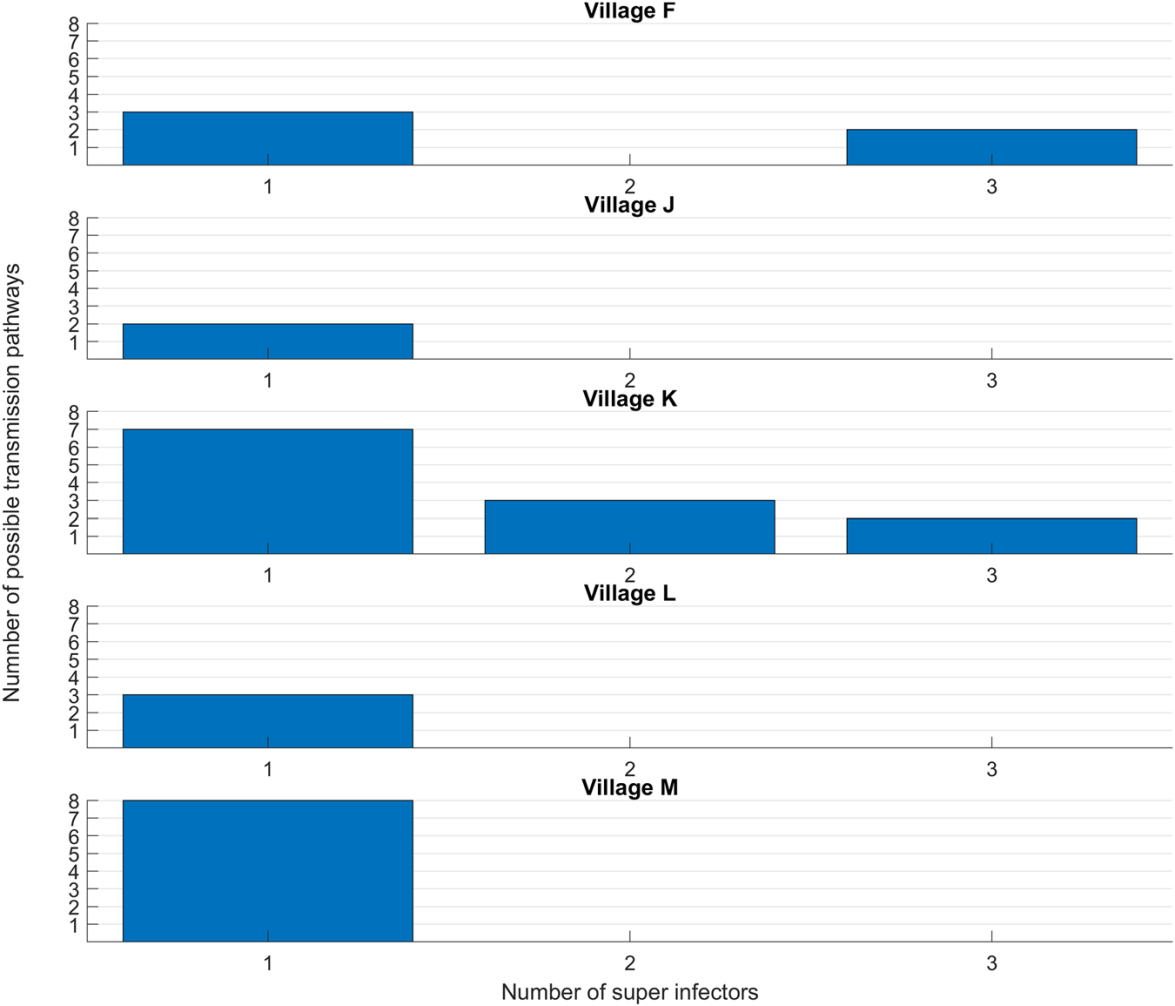
Distribution of multiple pathways from a single source infection (super-spreader) by village, in villages in eastern Gambia (J, K, L, and M) and one village in central Gambia (F). The x-axes show the number individuals (blue bars) from each village (labelled above each panel) identified as the source of two or more subsequent infections (y-axes).

To determine the presence of ‘super-infector’, i.e. barcodes related to those of at least two subsequent infections in travel/contact constrained paths, we relaxed the bi-directional optimization used to identify maximum likelihood pathways. There were 13 super-infectors who could have been the source of at least 2 subsequent infections (Fig. 6). The highest ‘super-infector’ was linked to 8 possible infections. The higher transmission sites in the east, especially village K in the south bank, had 46% of individuals likely to be the source of at least 2 or more infections.

## Discussion

Genotyping malaria parasites to determine relatedness between clones and assessing the temporal and spatial relationship among them has become a standard tool in malaria epidemiological research^15^. Such analysis can guide malaria control efforts by identifying genetically isolated *Plasmodium falciparum* populations, clusters of genetically similar clones maintaining local malaria transmission, those originating from outside a local transmission area or responsible for new local outbreaks in non-malaria regions^14,15^. We analysed variation across a panel of 54 SNPs in 355 *P*. *falciparum* infections, to characterise local populations and detect transmission paths across The Gambia. As previously shown in neighbouring Senegal, infection complexity was heterogeneous and lowest in the low transmission western regions close to the Atlantic coast^1,12^. Urbanisation in the western regions and probably the dominance of a less effective vector, *Anopheles melas*, contribute to the overall low transmission at these sites around salty river water or the Atlantic Ocean^16^. These low transmission regions included those with isolates having unique barcodes around the central regions and those with high levels of shared barcodes, like the more urban coastal towns. None or very few clinical malaria cases were reported in sites with unique *P*. *falciparum* barcodes^1^, where the isolates genotyped may include long-standing residual infections, which are not highly transmissible and not leading to clinical disease (at least some of them)^17,18^. Identifying and determining the presence of such infections is vital for malaria elimination and requires improved diagnostic approaches for targeted intervention. On the other hand, sites with multiple shared barcodes and highly similar parasite pairs in paired villages suggest clonal expansion similar to outbreaks after the introduction of infection(s). Such situations would require approaches able to detect and target infections “imported” into The Gambia, which is surrounded by Senegal, where there is still relatively high transmission in the southern-eastern regions^19^. These findings are interesting given that the overall village-scale analysis showed clustering of highly similar barcodes at household level, where the risk of clinical malaria is higher in households with at least one infected individual at the beginning of the transmission season^1^. Therefore, approaches such as reactive household interventions with drugs or other tools may target this infection reservoir and facilitate local elimination of malaria. Such interventions are already being tried and results may help further clarify our findings^20^. Further establishing the chain of transmission of such imported or local outbreaks could facilitate approaches towards elimination.

Transmission analysis can make use of genetic polymorphisms to link infectious pathogen isolates using genetic distance and phylogenetic approaches as applied for viruses and bacteria^21^. Analyses can be further constrained by known case-contact information and temporal-spatial distances to refine the likelihood of a transmission event between infection pairs. As previously shown in Kenya and The Gambia, variation between parasite genotypes increases with both time and geographical distance^4^. Intuitively, the likelihood of a transmission event should increase with decrease genetic distance between samples. However, direct application of genetic distances to detect malaria transmission events can be affected by several factors including; human migration, mutation and recombination of *P*. *falciparum* isolates from different geographical areas^22^. Transmission paths can be inferred by eliminating these evolutionary confounders of relatedness using probabilistic models of genetic distance within the linear range of the relationship with evolutionary distance^22^. Here we employed genetic distances within 30% (the linear range of correlation with evolutionary distance), reducing uncertainties in ancestry due to recombination. Moreover, Hudson’s measure of identity-by-descent^23^ correlated linearly within these genetic distances, increasing confidence in connectivity and recent ancestry between pairs in detected transmission paths^5^. Our novel model also combined two unobserved processes; within-host and within-vector parasite dynamics and duration of infection, with chronology of sampling and travel history of sampled individuals. Transmission paths were only restricted to sample pairs (source and infected) whose chronology satisfied the parasite’s life cycle. This enabled the estimation of the direction of *P*. *falciparum* (a recombining eukaryotic infectious pathogen) flow across The Gambia. More than half of the identified person-to-person transmission pathways occurred within household and village, indicating substantial local transmission. Although some infections in the east may have originated from western Gambia, there was a higher flux of parasites from east to west, particularly at peak transmission. Therefore, interrupting transmission in western Gambia would require intensifying control efforts in eastern Gambia and probably southern and eastern Senegal, where malaria transmission is higher^19^.

The analysis here was limited by the relatively low proportion of samples successfully genotyped, especially during the early months of the transmission season and from the low transmission peri-urban regions near the Atlantic coast of The Gambia. Thus, latent transmission links could have been missed from low parasite density samples and missing genotypes within transmission chains. Future genotyping studies can be improved by higher resolution sampling and inclusion of genome amplification steps as demonstrated for whole genome sequencing^24^. These can further apply recent *de novo* genomic variants approaches to reconstruct transmission trees^25^. This new whole genome approach remains largely inapplicable to most African malaria settings because of the higher complexity of infections and the poor availability of genome sequences from dense temporal and spatial samples. These difficulties explain the small number of studies investigating the relatedness and transmission pathways between malaria infections, either at small or large geographical scale in sub-Saharan Africa. Thus, SNP barcodes will continue to provide cheaper and low-density data which can be combined with our approach for transmission studies.

## Conclusions

We have shown a strong spatial hierarchy between genetic relatedness of pairs of *P*. *falciparum* isolates within households and villages, and between pairs of villages in close proximity. While this represents the local transmission hierarchy, long range transmission from high to low malaria prevalence areas were also detected. Hence, genotyping *P*. *falciparum* isolates can be used to track pathways of infection, including discriminating between indigenous and imported infections. It would be helpful to start considering a more programmatic use of these and other transmission tracking approaches in support of national malaria control programs to better target control efforts.

## Methods

### Study sites and samples

Data were collected as part of a study carried out between June 2013 and April 2014 in six pairs of villages across The Gambia^1^. Six sites, each comprising a pair of villages, were selected. Each village pair was within 5 km and differed by prevalence of infection according to a survey carried out in November 2012^18^. Each village was coded with a letter, from A to M (Fig. 1). Monthly blood samples (Whatman filter paper blood spots) were systematically collected from all consenting residents between June and December 2013. In addition, a blood sample was collected from suspected clinical malaria cases.

### Genotyping summary population genetic statistics

DNA was extracted from 3 discs of dried blood spots on filter paper using automated DNA extraction system (Qiagen). *Plasmodium spp* infection was tested by diagnostic and speciation PCR^26,27^. *P*. *falciparum* positive samples were genotyped for 86 SNPs (Supplementary Table 1) on the Sequenom MassARRAY iPLEX platform^28^ at the Broad Institute, Boston, USA^29^. Genotyping assays followed standard amplification and MALDI-TOF mass spectrometry and allele calling pipeline. Following assay quality control, SNP calls were made based on assay intensity values (r) and allelic signal intensity ratios (theta). Alleles were either classified as homozygous with the reference (0), alternative (1), or heterozygous (intermediate values). The multilocus SNP genotype calls were aggregated for all samples and filtered to remove loci with less than 25% call rate and per sample missingness of more than 12 loci (22%). The number of retained SNPs from chromosomes 1–14 were 2, 4, 3, 6, 1, 2, 5, 4, 3, 2, 4, 2, 7, 9, respectively. The filtered dataset with maximum loci missingness of 22% included 355 subjects (32% of samples attempted) and 54 SNPs (62.7% of 86 SNPs), (Supplementary Table 2). The most densely genotyped villages occurred in the east, in October and November (Supplementary Figs 1 and 2).

In case of mixed barcodes, the complexity of infection was determined following the model in McCOIL^30^. A genotype file representing the major alleles at mixed positions was generated and used to derive summary population genetic statistics (allele frequencies, observed and expected heterozygosity, multilocus linkage allelic association) in R statistical software (version 3.3.2). Allele frequency differences represented by average Fst over all pairs of villages was calculated with the hierFstat package. All further analyses for transmission paths were based on the “shared variant”. We retained the rarer alleles (minor variant) for isolates where loci had mixed calls. This approach considers that the presence of less common variants in same physical position between 2 barcodes maximise the likelihood of link between the infection pairs and offer additional resolution in detecting true transmission paths^31^. Compared to alternative approaches in which the major variants or random choice of allele at mixed positions are used, this did not significantly bias allele frequency spectra and genetic distances between pairs of isolates.

### Genetic barcode relatedness between *P*. *falciparum* infections

Genetic distance between isolate pairs was measured as p-distance, i.e. the number of substitutions (differences in identity by state) between two genotypes, expressed as the percentage of the number of SNP positions with alternative alleles between pairs of barcodes for all positions without missingness in the sample pair. Additionally, relatedness was inferred by calculating barcode identity by descent (IBD) using the hmmIBD algorithm^23^. Spatial distances were calculated as the geographic distance between sample sources (Km), based on their recorded latitude and longitude position. As The Gambia is uniformly low-lying, altitude was not considered in calculation of geographic distances. Temporal distance was expressed as the difference in days between sample collection dates. For all pairwise genetic distances, spatial and temporal differences were visualized as a 3D plot (Supplementary Fig. 5). The spatial and temporal hierarchical structure of the study design was evident.

### Inferring transmission events

We applied a model that quantifies the likelihood of a genetic distance between pairs of infections, while accounting for the time between the pairs. As the true date of infection was unknown for sampled populations, the sampling date was used as a proxy. The primary objective was to identify the most likely person-to-vector-to-person transmission (referred thereafter as person-to-person) paths and the direction from source to infected. These paths were constrained by the unobserved parasite life cycle time within the vector and length of infection between human sampling times (Fig. 3). In the pathway between samples in a transmission link, T_A_ represents the human infectious time since the previous sampling (Fig. 3A). It is assumed that individuals were bitten b days before they were sampled and genotyped. T_V_ and T_d_ represent the time for the vector to become infectious and to infect an uninfected individual, respectively. Their combination is constrained by the lifespan of the vector. After the infective bite, there are T_i_ days for the infection to appear in the peripheral blood and T_B_ days until a blood sample is collected to be genotyped. The observation-time difference between genotypes was therefore determined as; T_V_ + T_d_ + T_i_ + T_B_ − b, assumed to represent the evolutionary time between pairs of genotypes in a transmission path. Pairs of individuals may be linked to a common parent infection from the same vector or different vectors, with the observed genotypes only evolving from infection to observation-times. In this case, the duration between observation-times is T_d_ + T_i_^B^ + T_B_ − T_i_^A^ + T_A,_ assumed to be the evolutionary time between genotypes (Fig. 3B). Considering the monthly sampling, T_A_ and T_B_ are assumed to vary between 0 and 30 days and b to vary between 0 and T_A_. T_A_ and T_B_ were simulated from uniform distributions with a range of 0–30 days. T_V_ and T_i_ were simulated from triangular distributions, with ranges of 8–15 days and a mode of 11.5 days, and of 5–15 days with a mode of 10 days, respectively, representing the time range for an infected mosquito to develop sporozoites and transmit to an uninfected individual. The vector lifespan was simulated as a triangular distribution with range of 18–24 days and a mode of 21 days, with T_d_ simulated as lifespan – T_v_. Each model was simulated through 100,000 random sampling from the possible between-event time distributions. The distributions showed a clear overlap of the smoothed density of pairwise events for the same source and for the person-to-person transmission paths models. To increase the chance of identifying ‘who infected who’, the observation times between related genotypes were restricted to lie to the right of the 97.5% of the times for same source distributions (Supplementary Fig. 8). Thus, a range of 32 to 67 days are required between samples to observe a ‘directional’ transmission event.

SNP genotypes of 355 isolates were fitted with 88 possible evolutionary models using a maximum likelihood approach in IQ-TREE. The TVM (transversion model) + I (invariant sites) + G4 (4 regions of variable substitution rate) was the most appropriate model by the Bayesian Information Criterion. The evolutionary distances between all pairs of isolates for the optimal tree were calculated. The evolutionary distance saturated at a genetic distance of approximately 0.3 (p-distance adjusted for missingness), (Supplementary Fig. 7). For pairs of isolates with a genetic distance less than 0.3, the substitution rates were estimated by dividing the evolutionary distance with their observational time difference, assuming the time between source and recipient infection genotypes satisfied the person-to-person time constraint of at least 32 days. Ten thousand random barcodes of 54 SNPs were then evolved over 32 to 67 days via an MCMC model with TVM + I + G4 parameters from the optimal model with a bootstrapped sample of the estimated substitution rates. This resulted in a joint probability density (smoothed via 2-dimensional kernel density estimation) of genetic distance and the sampling time difference between isolates (Supplementary Fig. 9). For a given time between observed infections, the bivariate distribution was used to estimate the probability of the corresponding genetic distance between sample pairs. If the genetic distance exceeded 0.3, the transmission probability was assigned a zero-value due to the non-uniqueness of relationship between evolutionary and genetic distance. This limits the effect of unexplained genetic distances on the transmission chain inferred. Additionally, only sample pairs with a pairwise (and/or) missingness of 12 or less SNPs were considered in transmission paths. Under these conditions, there was no evidence of missingness biasing the p-distance estimates of genetic distance.

To improve the accuracy of the modified evolutionary model, it was additionally constrained by travel information within 30 days before sampling, thus precluding inter-village pathways for individuals who did not travel. With a sampling interval of 30 days or greater, the potential source subjects were restricted to travel within the previous 30 days. As the time from biting to detectable infection is greater than 30 days, the recipient subjects were constrained to have travelled within the last 60 days. For subject pairs with timing and travel constraint (i.e. at least one of the pairs must have travelled) satisfied, the bivariate density was used to ascribe a probability to each possible transmission pair based on the observed genetic and temporal difference. The probability of a transmission event was zero for pairs with a predicted genetic distance outside the 95% confidence limits of the bivariate distribution. Pairwise probabilities were weighted by the probability of an observed temporal difference (based on the life cycle simulation), given that the genotype pair arose from a person-to-person transmission. A transmission probability matrix between each pair of isolates was built in this manner. To avoid ambiguity in source versus recipient pathways and vice-versa, the most likely transmission paths were chosen as the pairs with the highest probability from a maximization over columns in each row and over rows in each column of the transmission probability matrix. This resulted in a set of pathways where each source had a unique maximum likelihood for an infected subject and vice-versa. Sources with more than 2 paths to subsequent infections were classified as ‘super-infectors’ (Supplementary Fig. 10), obtained by optimising over the row space (sources) only.

### Statistical analyses

Packages in R (version 3.3.2) were used to draw maps and calculate summary population genetic statistics. Evolutionary models and maximum likelihood phylogenetic trees were fitted using IQTREE (version 1.5.3). MCMC simulations, graphics and modelling of evolution and transmission likelihoods were performed in MATLAB (version R2016b).

### Ethical approval

Written informed consent was obtained from all participants ≥18 years old and parents/caregivers/guardians provided written informed assent for children aged 12–17 years following standardised good clinical research practice SOPs at MRCG-LSHTM. The study and protocols were approved by the Gambia Government/MRC Joint Ethics Committee (SCC 1318). All methods in this study were performed in accordance with the relevant guidelines and regulations enforce by the local EC and the MRCG-LSHTM research governance.

## Supplementary information

Supplementary figures

Supplementary table 1

Supplementary table 2

## Data Availability

Multilocus SNP genotypes for *Plasmodium falciparum* isolates are available in Supplementary Table 2.

## References

[1] Mwesigwa J (2017). Residual malaria transmission dynamics varies across The Gambia despite high coverage of control interventions. PLoS One.

[2] Niang M (2017). Substantial asymptomatic submicroscopic Plasmodium carriage during dry season in low transmission areas in Senegal: Implications for malaria control and elimination. PloS one.

[3] Stoddard ST (2009). The role of human movement in the transmission of vector-borne pathogens. PLoS neglected tropical diseases.

[4] Omedo I (2017). Micro-epidemiological structuring of *Plasmodium falciparum* parasite populations in regions with varying transmission intensities in Africa. Wellcome Open Res.

[5] Taylor AR (2017). Quantifying connectivity between local *Plasmodium falciparum* malaria parasite populations using identity by descent. PLoS Genet.

[6] Volkman SK (2012). Application of genomics to field investigations of malaria by the international centers of excellence for malaria research. Acta tropica.

[7] Daniels R (2008). A general SNP-based molecular barcode for *Plasmodium falciparum* identification and tracking. Malar J.

[8] Baniecki ML (2015). Development of a single nucleotide polymorphism barcode to genotype Plasmodium vivax infections. PLoS neglected tropical diseases.

[9] Preston MD (2014). A barcode of organellar genome polymorphisms identifies the geographic origin of *Plasmodium falciparum* strains. Nat Commun.

[10] Coll F (2014). A robust SNP barcode for typing Mycobacterium tuberculosis complex strains. Nature communications.

[11] Poon LL (2016). Quantifying influenza virus diversity and transmission in humans. Nature genetics.

[12] Daniels RF (2015). Modeling malaria genomics reveals transmission decline and rebound in Senegal. Proceedings of the National Academy of Sciences of the United States of America.

[13] Sisya TJ (2015). Subtle changes in *Plasmodium falciparum* infection complexity following enhanced intervention in Malawi. Acta tropica.

[14] Obaldia N (2015). Clonal outbreak of *Plasmodium falciparum* infection in eastern Panama. The Journal of infectious diseases.

[15] Koepfli C, Mueller I (2017). Malaria Epidemiology at the Clone Level. Trends in parasitology.

[16] Caputo B (2008). Anopheles gambiae complex along The Gambia river, with particular reference to the molecular forms of An. gambiae s.s. Malar J.

[17] Okebe J (2014). School-based countrywide seroprevalence survey reveals spatial heterogeneity in malaria transmission in the Gambia. PloS one.

[18] Mwesigwa J (2015). On-going malaria transmission in The Gambia despite high coverage of control interventions: a nationwide cross-sectional survey. Malar J.

[19] Thwing J (2017). Declines in Malaria Burden and All-Cause Child Mortality following Increases in Control Interventions in Senegal, 2005–2010. Am J Trop Med Hyg.

[20] Okebe J (2018). Reactive community-based self-administered treatment against residual malaria transmission: study protocol for a randomized controlled trial. Trials.

[21] Worby CJ, Lipsitch M, Hanage WP (2014). Within-host bacterial diversity hinders accurate reconstruction of transmission networks from genomic distance data. PLoS computational biology.

[22] Worby CJ (2016). Reconstructing transmission trees for communicable diseases using densely sampled genetic data. The annals of applied statistics.

[23] Schaffner SF, Taylor AR, Wong W, Wirth DF, Neafsey D (2018). E. hmmIBD: software to infer pairwise identity by descent between haploid genotypes. Malar J.

[24] Oyola SO (2016). Whole genome sequencing of *Plasmodium falciparum* from dried blood spots using selective whole genome amplification. Malar J.

[25] Redmond SN (2018). *De Novo* Mutations Resolve Disease Transmission Pathways in Clonal Malaria. Mol Biol Evol.

[26] Snounou G (1993). High sensitivity of detection of human malaria parasites by the use of nested polymerase chain reaction. Molecular and biochemical parasitology.

[27] Padley D, Moody AH, Chiodini PL, Saldanha J (2003). Use of a rapid, single-round, multiplex PCR to detect malarial parasites and identify the species present. Annals of tropical medicine and parasitology.

[28] Gabriel, S., Ziaugra, L. & Tabbaa, D. SNP genotyping using the Sequenom MassARRAY iPLEX platform. *Current protocols in human genetics* Chapter 2, Unit 2 12, 10.1002/0471142905.hg0212s60 (2009). 10.1002/0471142905.hg0212s60 19170031

[29] Gabriel, S., Ziaugra, L, Tabbaa, D SNP genotyping using the sequenom MassARRAY iPLEX platform. *Current protocols in human genetics* (2009). 10.1002/0471142905.hg0212s60 19170031

[30] Chang HH (2017). THE REAL McCOIL: A method for the concurrent estimation of the complexity of infection and SNP allele frequency for malaria parasites. PLoS computational biology.

[31] Worby CJ, Lipsitch M, Hanage WP (2017). Shared Genomic Variants: Identification of Transmission Routes Using Pathogen Deep-Sequence Data. Am J Epidemiol.

