# Supplementary material for: Long-distance transmission patterns modelled from SNP barcodes of Plasmodium falciparum infections in The Gambia
Source: Sci Rep. 2019 Sep 18;9:13515. doi: 10.1038/s41598-019-49991-4 (PMC6751170; doi:10.1038/s41598-019-49991-4)

Long-distance transmission patterns modelled from SNP barcodes of *Plasmodium falciparum* infections in The Gambia

Alfred Amambua-Ngwa[1*], David Jeffries[1], Julia Mwesigwa[1], Aminata Seedy-Jawara[1], Joseph Okebe[1], Jane Achan[1], Chris Drakeley[3], Sarah Volkman[2], Umberto D'Alessandro[1,3]

1. Medical Research Council Unit The Gambia at London School of Hygiene and Tropical Medicine
2. Harvard School of Public Health, Boston, Massachusetts, USA
3. London School of Hygiene and tropical Medicine, London, UK

*Correspondence to; angwa@mrc.gm, alfred.ngwa@lshtm.ac.uk

Supplementary figure 1. Map of The Gambia showing roads (traced in white) connecting sampling sites from West to East and on both banks of the river Gambia. The international boundaries of the Gambia are traced in red. Houses from which samples were successfully genotyped are marked with red points on zoom-in boxes of each village indicated on the map with red arrow. Map was created using Leaflet for R. Satellite image is the standard ARCGIS online imagery, sources: Esri, DigitalGlobe, GeoEye, i-cubed, USDA FSA, USGS, AEX, Getmapping, Aerogrid, IGN, IGP, swisstopo, and the GIS User Community. Map was produced on 29th May 2019; https://www.arcgis.com/home/webmap/viewer.html?url=https%3A%2F%2Fserver.arcgisonline.com%2Farcgis%2Frest%2Fservices%2FWorld_Imagery%2FMapServer&source=sd. ArcGIS version 10.3.1 (ESRI, Redlands, CA, USA) was used to map and visualize spatial positions of sites. Topographic data were obtained from DIVA-GIS Free Spatial Data (http://www.diva-gis.org/gdata) and ESRI (http://www.arcgis.com/home/item.html?id=30e5fe3149c34df1ba922e6f5bbf808f). Roads were obtained from Roads© OpenStreetMap contributors and available from https://www.openstreetmap.org through https://overpass-turbo.eu/

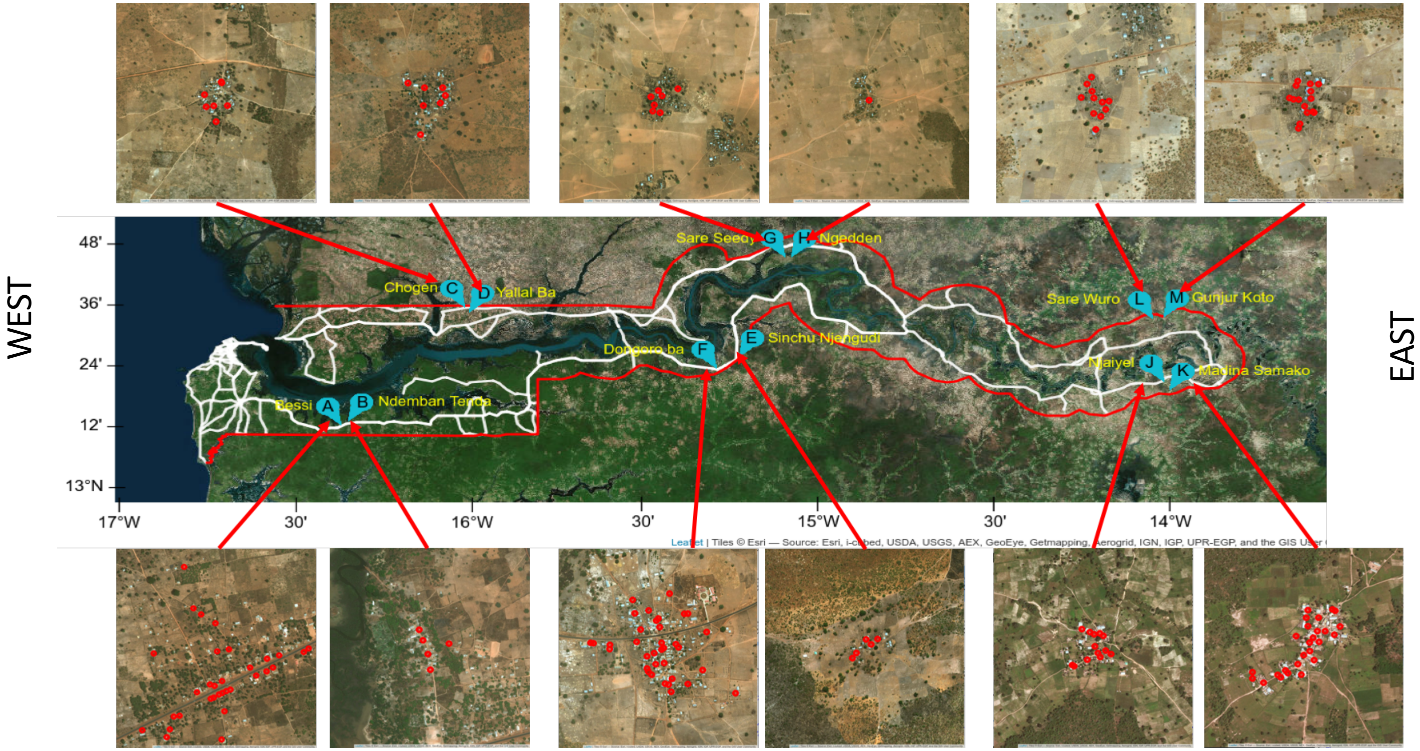

Supplementary figure 2: Distribution of samples and complexity of infection from July to December (y-axis) at sites from the Western, Central and Eastern regions (x-axis). Points are colour coded by the predicted clonality; single (COI 1) or at least 2 clones (a COI of two or more) in the infection.

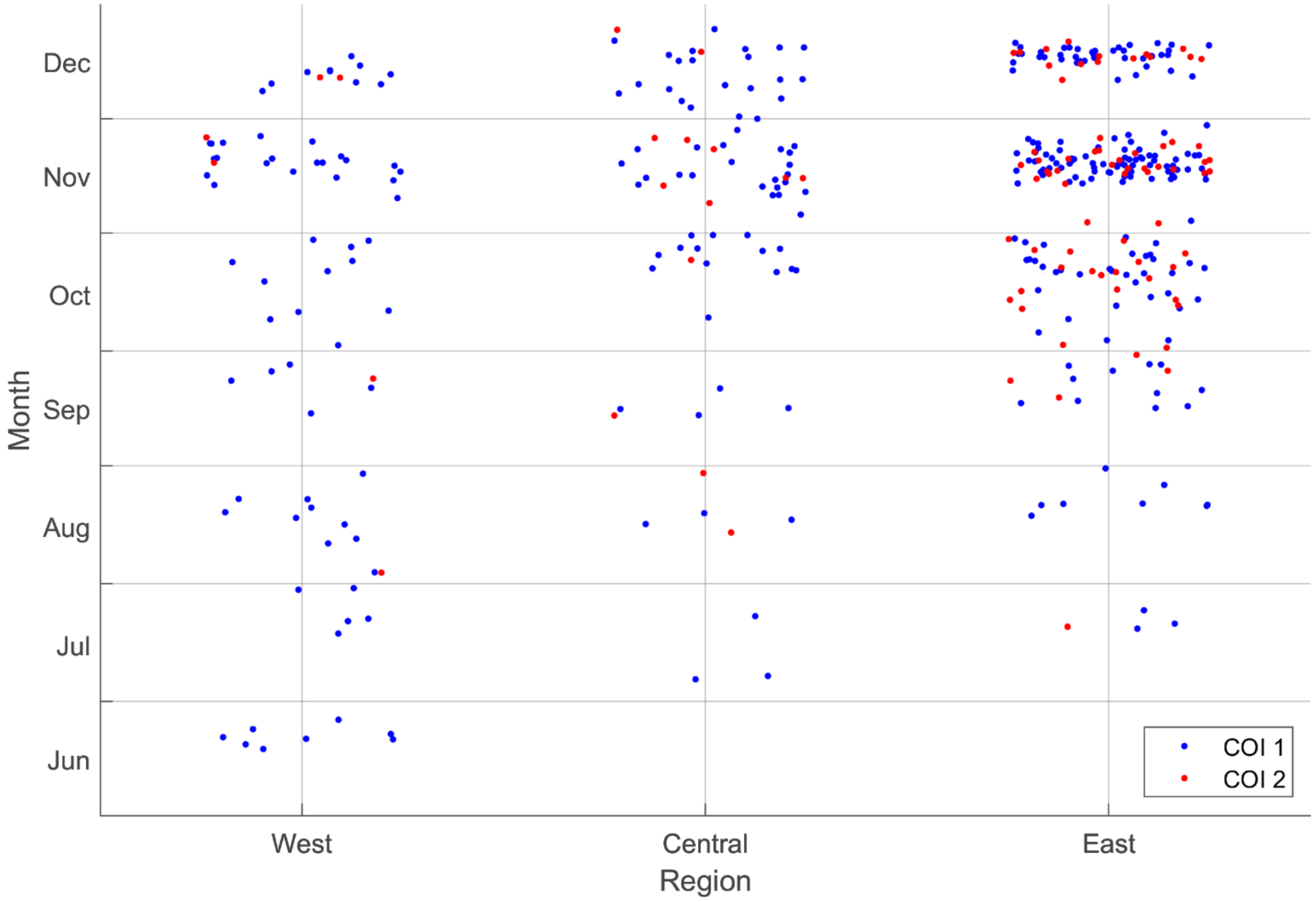

Supplementary figure 3. Heatmap of pairwise barcode similarities (1- genetic distance) between isolates from sampling sites (axes). a) All pairwise similarity heat-coloured as on the bar key. a) Heatmap of isolates with pairwise barcode similarities greater than 95%.

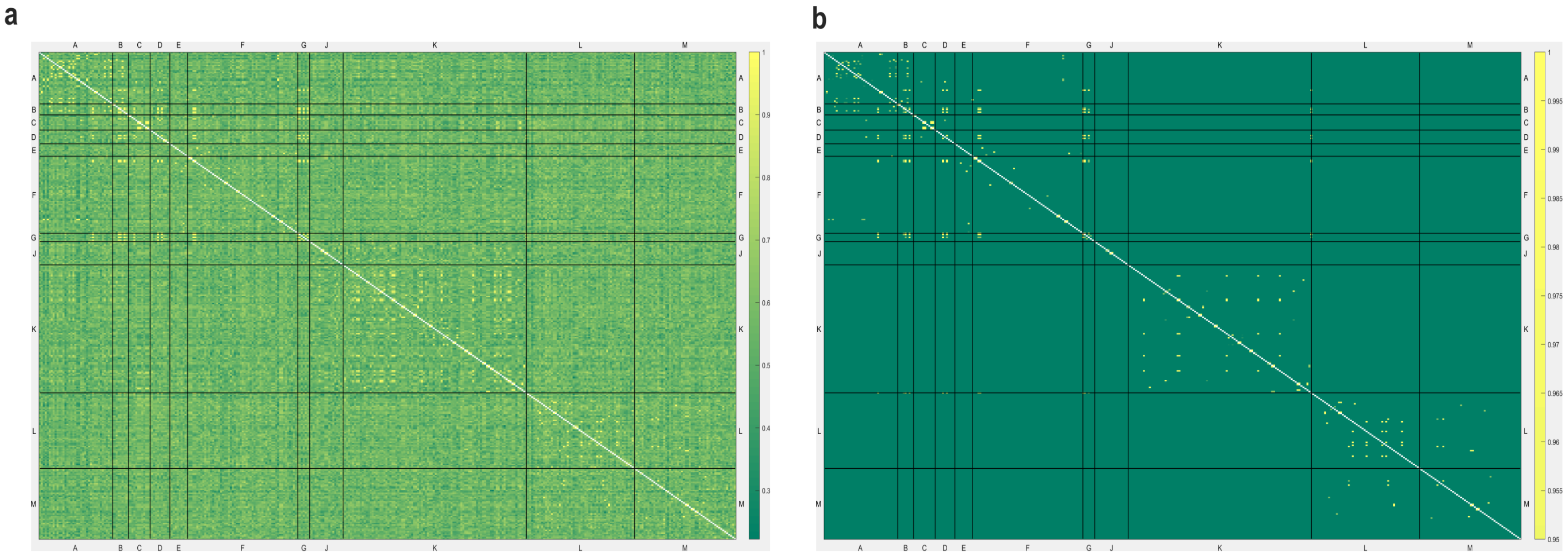

Supplementary Figure 4. All pairwise genetic distances between isolate barcodes separated by the sampling time difference between pairs. Each point (blue) represents a pair. The red line shows the trend in pairwise distances with change in number of days between samples.

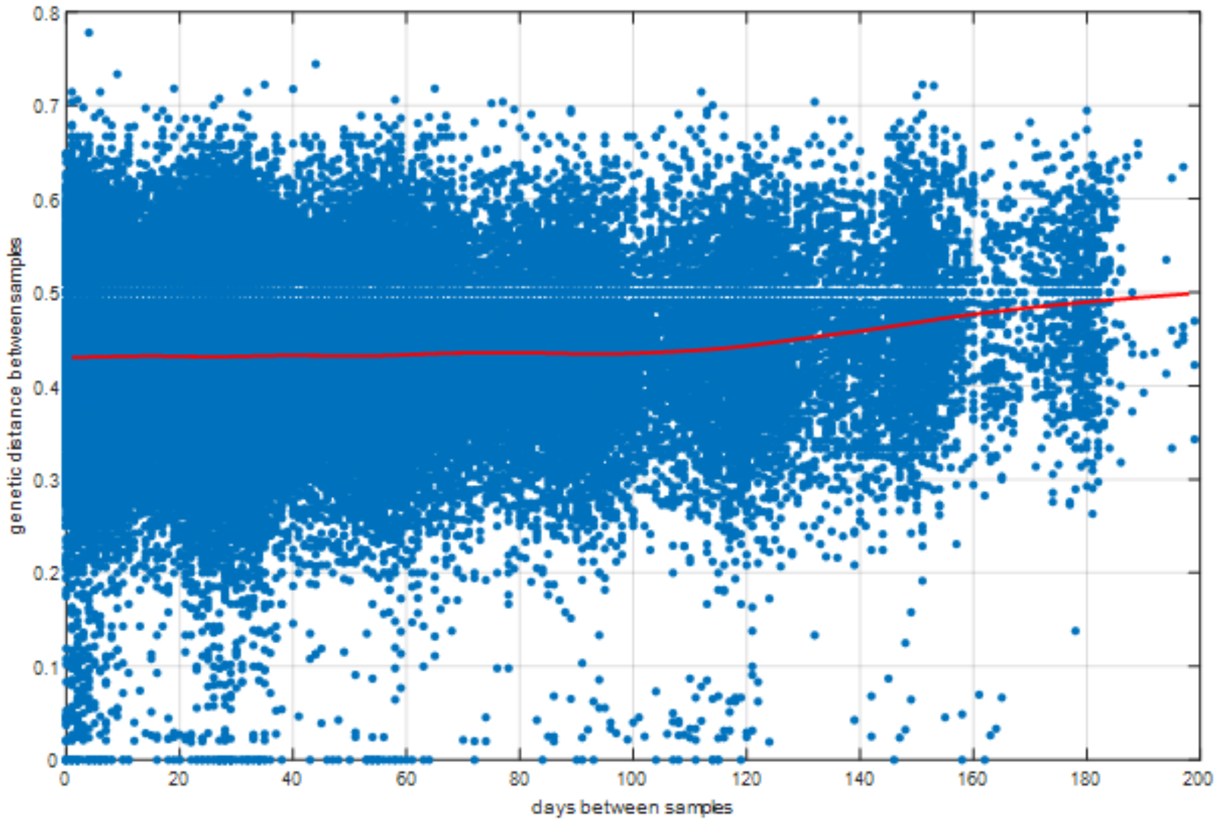

Supplementary figure 5. 3-way plot between temporal distance (days), geographic distance (km) and genetics distance. Each point (violet) represent the values for each pair of isolates.

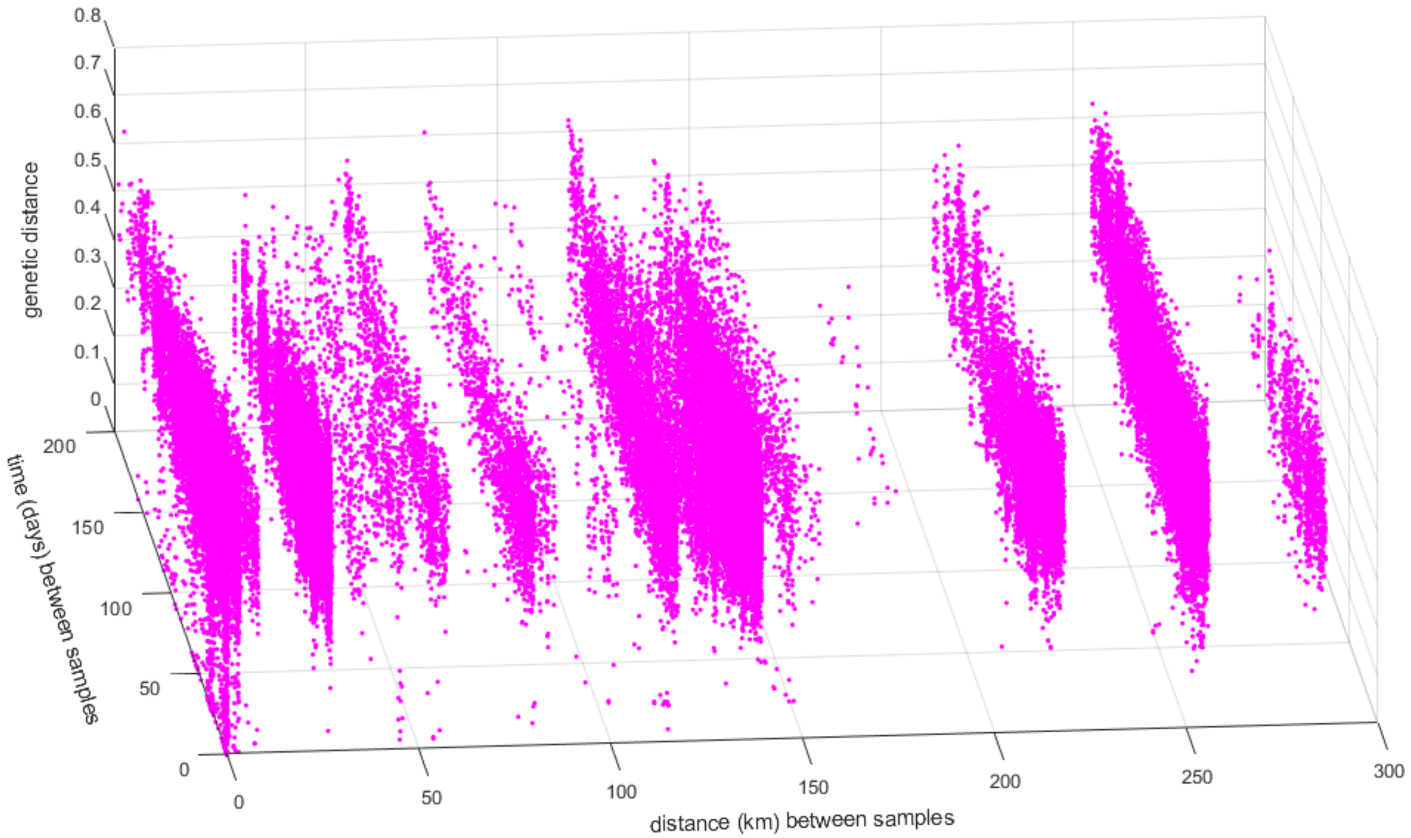

Supplementary figure 6. Pairwise identity-by-descent (IBD) between isolates and correlation with pairwise genetic distance. a) Heatmap of pairwise IBD of isolates across all sites coloured coded from 0 (low IBD) to 1 (high IBD) as indicated on the side bar. b) Correlation between identity-by-descent(IBD), (x-axis), and genetic distance (y-axis) determined from the SNP barcodes of 355 isolates from across The Gambia. Each point represents hmmIBD determined IBD plotted against the genetic distance (p-distance) for each isolate. The red line is a fitted cline of the distances showing saturation of linear relation between both measure of relatedness.

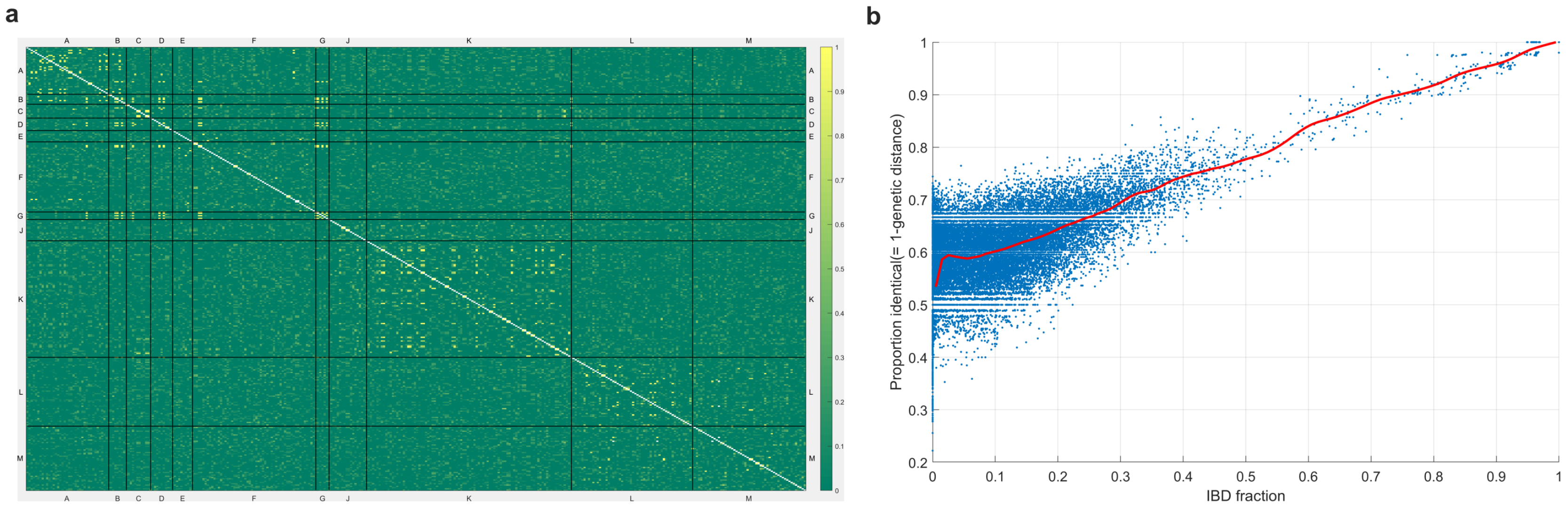

Supplementary figure 7. Correlation between evolutionary (x-axis) and genetic (y-axis) determined from the barcodes of 355 isolates from across The Gambia. Each point represent evolutionary distance (substitution per site) plotted against the genetic distance (p-distance) for each isolate. The red line is a fitted cline of the distances showing saturation of linear relation at a genetic distance of 0.3.

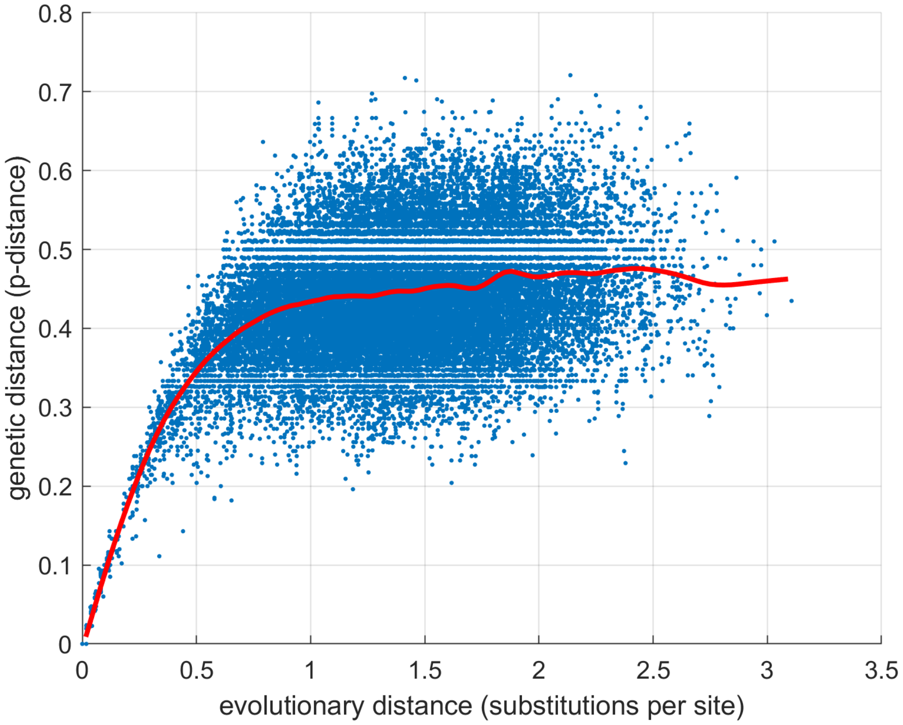

Supplementary Figure 8: Smoothed density distribution of the time between of pairwise events (transmission paths) for same source and person-to-person models of transmission.

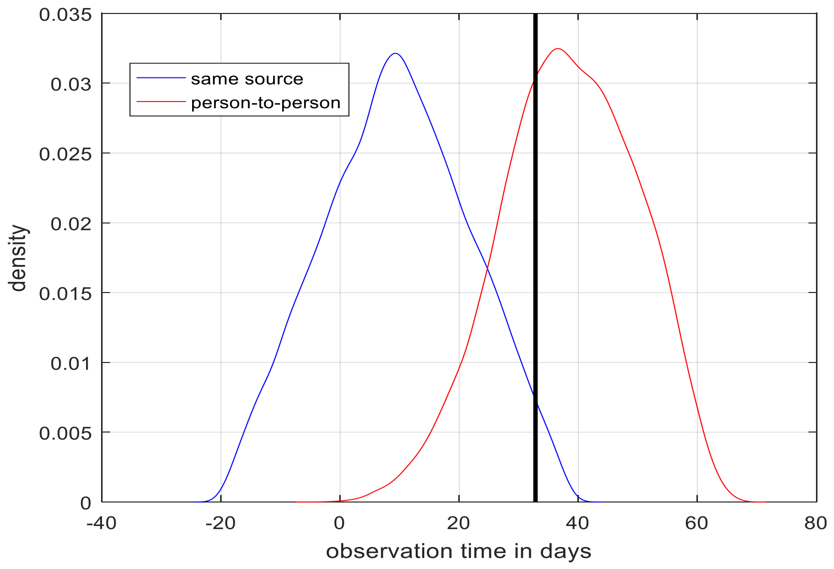

Supplementary figure 9: Joint probability density of genetic distance and the sampling time (days) between pairs of *P. falciparum* infected samples. The smoothed bivariate distribution was derived from simulation of the evolutionary model via the MCMC .

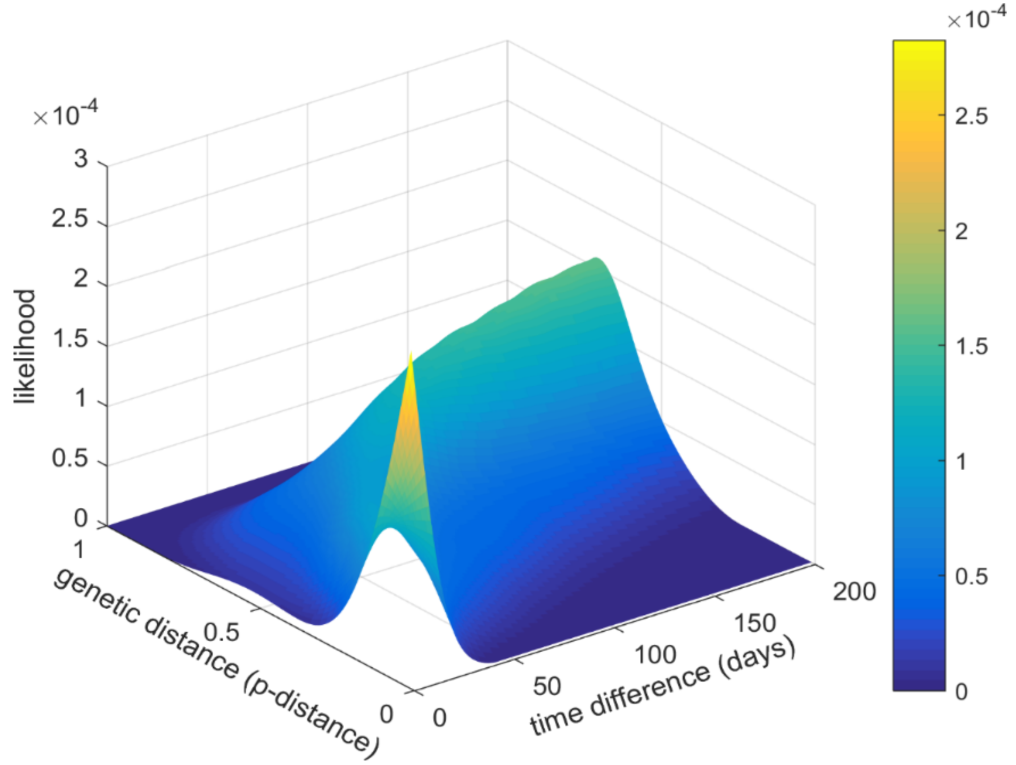

Supplementary figure 10. Histogram of the frequency of samples according to the number of pathways in which they are involved.

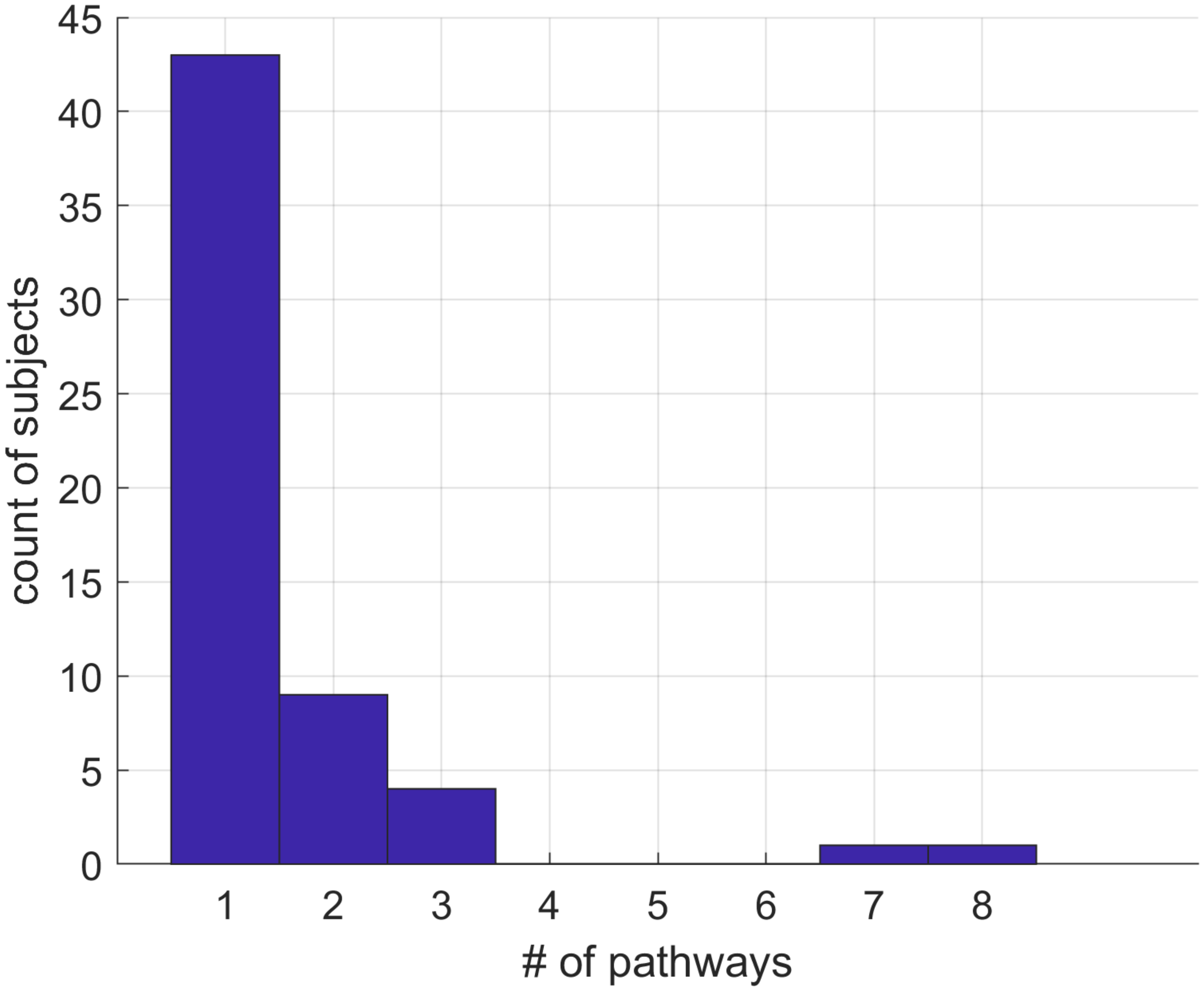

Supplement: Supplementary file 1 — Supplementary figures [file 41598_2019_49991_MOESM1_ESM.pdf]
